# Editorial: Development of the hypothalamus

**DOI:** 10.3389/fnana.2015.00083

**Published:** 2015-06-23

**Authors:** Gonzalo Alvarez-Bolado, Valery Grinevich, Luis Puelles

**Affiliations:** ^1^Department of Neuroanatomy, University of HeidelbergHeidelberg, Germany; ^2^Schaller Research Group on Neuropeptides, German Cancer Research Center and University of HeidelbergHeidelberg, Germany; ^3^Department of Human Anatomy and IMIB, University of MurciaMurcia, Spain

**Keywords:** circadian, MCH, Notch, oxytocin, prosomeric, Shh, thyroid, zebrafish

The hypothalamus is the region of the brain in charge of homeostasis as well as homeostatic behaviors like eating and drinking. The anatomical, connectional and physiological complexity of this region matches the importance and intricacy of its functions. Perhaps because of this, research on the developing hypothalamus has lagged behind that on the cortex or hippocampus. The realization that current pathological conditions like, e.g., some forms of obesity, hypertension and hormonal dysfunctions have its origin in developmental alterations of the hypothalamus has turned the focus again on this region. In the present Research Topic we try to give an idea of a variety of approaches, morphological, comparative and genetic, to key developmental questions related to the hypothalamus. In this collection, a focus on essential questions on the nature of this brain region and its specification is obvious.

We start logically with papers explaining how the hypothalamus can be subdivided. Differentially with all earlier monographs on the hypothalamus, we reflect here the increasing perception in the field that the classic columnar model is outdated and incompatible with accruing molecular data on the hypothalamus. Many workers are turning to the updated prosomeric model as an instrument for morphologic and causal interpretation. This model has been presented in several articles, and several progressive changes and improvements made to it (Puelles et al., 1987, 2012; Puelles and Rubenstein, 1993, 2003; Rubenstein et al., 1994; Puelles, 1995; Shimamura et al., 1995). The model proposes that the hypothalamus is the rostralmost part of the neural tube, since the developmental forebrain length axis ends in the terminal wall of the hypothalamus (i.e., the telencephalon is a dorsal outgrowth of the alar hypothalamus). The resulting peduncular and terminal hypothalamic subdivisions of the rostral part of the neural tube are admittedly divergent with those traditionally taught under the columnar viewpoint. The hypothalamus is perhaps the brain region undergoing the most puzzling changes in the model, and this curiously turns out to the advantage of causal explanations. For this reason, the place of the hypothalamus in the prosomeric model and corresponding molecular subdivisions were recently the subject of a long and scholarly book chapter (Puelles et al., 2012). This includes the so-called updated prosomeric model.

Here, Puelles and Rubenstein offer a more succint and very clear explanation of the changes introduced in their updated model, tracing their fundament to establish a solid basis of explicit reasonable assumptions, pointing out also some novel morphologic highlights, like the interpretation of the course of the fornix tract, or the newly-defined acroterminal domain (Puelles and Rubenstein, 2015). If this prosomeric paradigm is correct, gene expression patterns should support it. Therefore, the authors of the model mined the most extensive developmental gene expression database, the Allen Developing Mouse Brain Atlas (Allen-Institute-for-Brain-Science, 2009), in order to find additional support for the proposed Peduncular Hypothalamus, Terminal Hypothalamus, and Acroterminal Domain (Ferran et al., 2015). They find a number of early expression domains not only supporting the proposed subdivision, but also contributing to explain why certain structures are formed within certain domains.

The hypothalamus is a brain region having intimate implication in the life story of animal species: heat production, eating and drinking behavior, reproduction, all are regulated by it. For this reason the hypothalamus might be thought to be very species-specific. Are the gene expression patterns and distinct morphological fates on which the model is based specific of mammals? Or are they on the contrary ancestral to vertebrates and thus widely shared across different species, irrespective of evolutionary variation? To answer this question it was important to test the current prosomeric model of the hypothalamus, built mostly on observations on mammalian brains, in other vertebrate classes. Here we show two contributions in this direction. Domínguez et al. reviewed characteristic gene expression patterns (genoarchitectonic approach) in amphibians and in reptiles, and found shared traits as well as differences that underline the anamniote/amniote transition (Domínguez et al., 2015). Other workers focused similarly on the hypothalamus of an elasmobranch, the cat-shark, noting that a prosomeric organization is also found in cartilaginous fishes, so that it can be concluded that the model subdivisions are ancestral to jawed vertebrates (Santos-Durán et al., 2015).

Then we move on to the early steps in hypothalamic regionalization. One key gene in the prosomeric subdivision is Sonic hedgehog (Shh), and it could not be absent here. A concise review highlights the main characteristics of expression, progenitor domains and roles of Shh in hypothalamic development (Blaess et al., 2014). Shh signaling exerts its effects through the Gli family of transcription factors. These can act either as activators or repressors of downstream genes, and the balance between these opposite functions of the Gli proteins (the “Shh-Gli code”) is the basis of the effects of Shh on differentiation. Earlier analyses have unraveled the Shh-Gli code in the mouse spinal cord (see for instance Ericson et al., 1997; Ruiz i Altaba et al., 2003; Bai et al., 2004; Stamataki et al., 2005). Here, Haddad-Tóvolli et al. (2015) have approached the question of the particular roles of Gli2 and Gli3 as activators or repressors and their possible contribution to hypothalamic regional differentiation (the hypothalamic Shh-Gli code). As part of their analysis, the authors mapped their results onto the hypothalamus model, using it for the first time as a tool of phenotype analysis. Among other novel results, this study shows that hypothalamic regional diversity depends on differentially stringent requirements for Gli2 and Gli3. Ware et al. (2014) then review the available evidence on early hypothalamic neurogenesis. They conclude that a genetic network including members of the Notch pathway as well as proneural transcription factors is essential for the regulation of the initial neurogenesis occurring within the SHH-controlled hypothalamic basal plate.

The zebrafish is a key animal model to understand early hypothalamus development. The advantages of the zebrafish as developmental model system are well-known: embryos are very abundant, are transparent and develop rapidly and outside the mother. This makes it possible to physically manipulate them as well as live-imaging complex morphogenetic processes. Neural development studies, and in particular approaches to the early specification of regions like the hypothalamus, have benefited from the ease and speed of the genetic analysis in this model, for instance through gene knockdown or overexpression as well as the generation of transgenic fish (carrying inheritable genetic alterations). This has made the zebrafish model particularly useful to uncover gene function. As a result of the sequencing of the zebrafish genome, we know that around 70% of human genes have zebrafish orthologues. Many of these orthologues are involved in basic processes of brain specification and patterning. Hundreds of zebrafish mutant phenotypes have been identified through large-scale genetic screens, including many resembling human pathological conditions. Finally, having another vertebrate model system besides mammals and birds (*Gallus gallus*) to study the developing brain adds to the richness and variety of our knowledge in the field of comparative neuroanatomy.

In this collection, three different contributions approach important questions by analyzing early patterning events in zebrafish mutants. Manoli and Driever (2014) investigated the role of the transcription factor genes *Nkx2.1*, *Nkx2.4a*, and *Nkx2.b* in hypothalamic specification. They show that these regulators have differential roles in the development of the preoptic, intermediate and caudal hypothalamic regions.

Analyzed the developmental expression of peptide genes in the. Herget et al. (2014) recently defined the “neurosecretory preoptic area” (NPO) as the zebrafish larval paraventricular nucleus. Here, Herget and Ryu (2015) generate a complex map of peptide gene expression of this region as the basis for approaches to the neuroendocrine hypothalamus in the developing zebrafish.

Biran et al. (2015) review evidence on embryonic and postnatal expression of regulatory genes in the hypothalamus of zebrafish and mouse. They find that a number of transcription factors important for correct development are later redeployed in order to regulate key adult functions. Diaz et al. (2014) use peptide expression to characterize progenitor domains in the early hypothalamic ventricular zone of the mouse, then carefully map the migration of specific peptide-expressing neuronal subpopulations to their final settling place. They find that intricate tangential and radial migration routes as well as a considerable degree of mixing between the original subpopulations underlie the complex adult anatomy of the hypothalamus.

The previously unsuspected prevalence of tangential migrations illuminates the complexity of cell typology in many hypothalamic areas, and raises the possibility of functional alterations due to abnormal migrations. These peptides indeed have important functions, and oxytocin has come to be seen as the model for all of them, given its importance in behavior. Valery Grinevich and his coworkers have reviewed the development of the central oxytocin system and its alteration in patients afflicted with neurodevelopmental diseases (such as Prader-Willi syndrome and autism spectrum disorders) and respective animal models (Grinevich et al., 2014). At the end of the article, the authors discuss the therapeutic use of oxytocin for treatment of deficits in psychosocial and affiliative behaviors.

Croizier et al. (2014) review the development of the major axonal tracts coursing through the hypothalamus. They find that one important source of orientation for the main projection pathways is the earliest generated hypothalamic mantle. Later, these pathways will guide the formation of other afferent and efferent tracts of the prosencephalon. Makarenko (2014) has collated the available data on the time schedule and navigation pathways of the main hypothalamic tracts in the rat brain. She elaborates a precise calendar of projection development, and finds that each of the tracts is characterized by very specific spatio-temporal parameters.

Alkemade (2015) reviews contributions showing that, although the fetus is dependent on thyroid hormone from the mother, it can locally regulate its concentration not only in serum but also in the hypothalamus. The interplay between hormone sources and modulatory mechanisms achieves the precisely timed maturation of the hypothalamus (not too early, not too late).

We cannot focus on the development of all hypothalamic nuclei, but approach here two particularly emblematic ones. Landgraf et al. (2014) succinctly review the development of the circadian clocks. They conclude that, although we have good knowledge of the anatomical formation of the suprachiasmatic nucleus, there is still considerable debate about the prenatal emergence of rhythmicity. Sanchez-Arrones et al. (2015) use experimental fate-mapping together with prosomeric and genoarchitectonic principles in order to elucidate the developmental origin of the adenohypophysis in the chicken.

Finally, the hypothalamus and its heterogeneity can be used as a model to investigate key brain development problems. Szabó et al. (2015) approached a neglected developmental problem, the specific aggregation of neurons to form neuronal nuclei, using the mammillary body as a model. The authors show that two information systems, based on birthdates and adhesion mechanisms, work together in order to guarantee appropriate neuronal aggregation as well as specific axonal fasciculation. Both systems are implemented on the basis of sequential and hierarchical functions of classical cadherins.

## Conflict of interest statement

The authors declare that the research was conducted in the absence of any commercial or financial relationships that could be construed as a potential conflict of interest.

## References

[B1] Alkemade A. (2015). Thyroid hormone and the developing hypothalamus. Front. Neuroanat. 9:15. 10.3389/fnana.2015.00015 25750617 PMC4335174

[B2] Allen-Institute-for-Brain-Science. (2009). Allen Developing Mouse Brain Atlas [Internet]. Available online at: http://developingmouse.brain-map.org

[B3] Bai C. B. Stephen D. Joyner A. L. (2004). All mouse ventral spinal cord patterning by hedgehog is Gli dependent and involves an activator function of Gli3. Dev. Cell 6, 103–115. 10.1016/S1534-5807(03)00394-0 14723851

[B4] Biran J. Tahor M. Wircer E. Levkowitz G. (2015). Role of developmental factors in hypothalamic function. Front. Neuroanat. 9:47. 10.3389/fnana.2015.00047 25954163 PMC4404869

[B5] Blaess S. Szabó N. Haddad-Tovolli R. Zhou X. Alvarez-Bolado G. (2014). Sonic hedgehog signaling in the development of the mouse hypothalamus. Front. Neuroanat. 8:156. 10.3389/fnana.2014.00156 25610374 PMC4285088

[B6] Croizier S. Chometton S. Fellmann D. Risold P. Y. (2014). Characterization of a mammalian prosencephalic functional plan. Front. Neuroanat. 8:161. 10.3389/fnana.2014.00161 25610375 PMC4285092

[B7] Diaz C. Morales-Delgado N. Puelles L. (2014). Ontogenesis of peptidergic neurons within the genoarchitectonic map of the mouse hypothalamus. Front. Neuroanat. 8:162. 10.3389/fnana.2014.00162 25628541 PMC4290630

[B8] Domínguez L. González A. Moreno N. (2015). Patterns of hypothalamic regionalization in amphibians and reptiles: common traits revealed by a genoarchitectonic approach. Front. Neuroanat. 9:3. 10.3389/fnana.2015.00003 25691860 PMC4315040

[B9] Ericson J. Briscoe J. Rashbass P. van Heyningen V. Jessell T. M. (1997). Graded sonic hedgehog signaling and the specification of cell fate in the ventral neural tube. Cold Spring Harb. Symp. Quant. Biol. 62, 451–466. 10.1101/SQB.1997.062.01.053 9598380

[B10] Ferran J. L. Puelles L. Rubenstein J. L. (2015). Molecular codes defining rostrocaudal domains in the embryonic mouse hypothalamus. Front. Neuroanat. 9:46. 10.3389/fnana.2015.00046 25941476 PMC4400913

[B11] Grinevich V. Desarménien M. G. Chini B. Tauber M. Muscatelli F. (2014). Ontogenesis of oxytocin pathways in the mammalian brain: late maturation and psychosocial disorders. Front. Neuroanat. 8:164. 10.3389/fnana.2014.00164 25767437 PMC4341354

[B12] Haddad-Tóvolli R. Paul F. A. Zhang Y. Zhou X. Theil T. Puelles L. (2015). Differential requirements for Gli2 and Gli3 in the regional specification of the mouse hypothalamus. Front. Neuroanat. 9:34 10.3389/fnana.2015.00034 PMC4373379 25859185

[B13] Herget U. Ryu S. (2015). Coexpression analysis of nine neuropeptides in the neurosecretory preoptic area of larval zebrafish. Front. Neuroanat. 9:2. 10.3389/fnana.2015.00002 25729355 PMC4325906

[B14] Herget U. Wolf A. Wullimann M. F. Ryu S. (2014). Molecular neuroanatomy and chemoarchitecture of the neurosecretory preoptic-hypothalamic area in zebrafish larvae. J. Comp. Neurol. 522, 1542–1564. 10.1002/cne.23480 24127437

[B15] Landgraf D. Koch C. E. Oster H. (2014). Embryonic development of circadian clocks in the mammalian suprachiasmatic nuclei. Front. Neuroanat. 8:143. 10.3389/fnana.2014.00143 25520627 PMC4249487

[B16] Makarenko I. G. (2014). DiI tracing of the hypothalamic projection systems during perinatal development. Front. Neuroanat. 8:144. 10.3389/fnana.2014.00144 25538571 PMC4255665

[B17] Manoli M. Driever W. (2014). nkx2.1 and nkx2.4 genes function partially redundant during development of the zebrafish hypothalamus, preoptic region, and pallidum. Front. Neuroanat. 8:145. 10.3389/fnana.2014.00145 25520628 PMC4251446

[B18] Puelles L. (1995). A segmental morphological paradigm for understanding vertebrate forebrains. Brain Behav. Evol. 46, 319–337. 10.1159/000113282 8564469

[B19] Puelles L. Amat J. A. Martinez-de-la-Torre M. (1987). Segment-related, mosaic neurogenetic pattern in the forebrain and mesencephalon of early chick embryos: I. Topography of AChE-positive neuroblasts up to stage HH18. J. Comp. Neurol. 266, 247–268. 10.1002/cne.902660210 3437075

[B20] Puelles L. Martinez-de-la-Torre M. Bardet S. Rubenstein J. L. R. (2012). Hypothalamus, in The Mouse Nervous System, eds Watson C. Paxinos G. Puelles L. (San Diego, CA: Elsevier-Academic Press), 221–312.

[B21] Puelles L. Rubenstein J. L. (1993). Expression patterns of homeobox and other putative regulatory genes in the embryonic mouse forebrain suggest a neuromeric organization. Trends Neurosci. 16, 472–479. 10.1016/0166-2236(93)90080-6 7507621

[B22] Puelles L. Rubenstein J. L. (2003). Forebrain gene expression domains and the evolving prosomeric model. Trends Neurosci. 26, 469–476. 10.1016/S0166-2236(03)00234-0 12948657

[B23] Puelles L. Rubenstein J. L. (2015). A new scenario of hypothalamic organization: rationale of new hypotheses introduced in the updated prosomeric model. Front. Neuroanat. 9:27. 10.3389/fnana.2015.00027 25852489 PMC4365718

[B24] Rubenstein J. L. Martinez S. Shimamura K. Puelles L. (1994). The embryonic vertebrate forebrain: the prosomeric model. Science 266, 578–580. 10.1126/science.7939711 7939711

[B25] Ruiz i Altaba A. Nguyen V. Palma V. (2003). The emergent design of the neural tube: prepattern, SHH morphogen and GLI code. Curr. Opin. Genet. Dev. 13, 513–521. 10.1016/j.gde.2003.08.005 14550418

[B26] Sanchez-Arrones L. Ferran J. L. Hidalgo-Sanchez M. Puelles L. (2015). Origin and early development of the chicken adenohypophysis. Front. Neuroanat. 9:7. 10.3389/fnana.2015.00007 25741242 PMC4330794

[B27] Santos-Durán G. N. Menuet A. Lagadec R. Mayeur H. Ferreiro-Galve S. Mazan S.. (2015). Prosomeric organization of the hypothalamus in an elasmobranch, the catshark Scyliorhinus canicula. Front. Neuroanat. 9:37. 10.3389/fnana.2015.00037 25904850 PMC4389657

[B28] Shimamura K. Hartigan D. J. Martinez S. Puelles L. Rubenstein J. L. (1995). Longitudinal organization of the anterior neural plate and neural tube. Development 121, 3923–3933 8575293 10.1242/dev.121.12.3923

[B29] Stamataki D. Ulloa F. Tsoni S. V. Mynett A. Briscoe J. (2005). A gradient of Gli activity mediates graded Sonic Hedgehog signaling in the neural tube. Genes Dev. 19, 626–641. 10.1101/gad.325905 15741323 PMC551582

[B30] Szabó N. E. Haddad-Tóvolli R. Zhou X. Alvarez-Bolado G. (2015). Cadherins mediate sequential roles through a hierarchy of mechanisms in the developing mammillary body. Front. Neuroanat. 9:29. 10.3389/fnana.2015.00029 25852491 PMC4365714

[B31] Ware M. Hamdi-Rozé H. Dupé V. (2014). Notch signaling and proneural genes work together to control the neural building blocks for the initial scaffold in the hypothalamus. Front. Neuroanat. 8:140. 10.3389/fnana.2014.00140 25520625 PMC4251447

